# Cost of hospitalization and length of stay of hypoglycemic events in hospitalized patients with diabetes mellitus: A cross-sectional study

**DOI:** 10.1097/MD.0000000000041840

**Published:** 2025-03-14

**Authors:** Abdallah Y. Naser, Sami Qadus, Hind M. AlOsaimi, Abdulrahman AlFayez, Haya Bin Huwayshil, Lujain A. Al Harbi, Malak S. Alqhtani, Nayef A. Alyamani

**Affiliations:** a Department of Applied Pharmaceutical Sciences and Clinical Pharmacy, Faculty of Pharmacy, Isra University, Amman, Jordan; b Department of Pharmacy, Faculty of Health Sciences, American University of Madaba, Madaba, Jordan; c Pharmacy Services Administration at King Fahad Medical City, Riyadh Second Health Cluster, Riyadh, Saudi Arabia.

**Keywords:** cost, diabetes mellitus, economic, hospitalization, hypoglycemia

## Abstract

This study aims to assess the length of stay and cost per hypoglycemia episode, as well as to determine the factors that influence the length of stay, intensive care unit (ICU) admission, and hospitalization costs among patients with diabetes mellitus. This is a retrospective cross-sectional study conducted on a cohort of diabetic individuals who experienced confirmed hypoglycemia episodes. The data pertaining to these patients were obtained from their respective hospital medical records, covering the period from January 2021 to December 2022. King Fahd Medical City was selected as the site of data collection for this study. A total of 396 patients were involved in this study. The median duration of stay for the patients was 7.0 (2.0–16.0) days. Only 3.0% of the patients had a previous hypoglycemia admission history. Around 53.3% of the patients were admitted to the ICU. The median duration of ICU admission stay was 1.0 (0.0–1.0) days. The highest cost driver for patients with hypoglycemia was ICU stay with a median cost of 9000.0 (1125.0–15750.0) Saudi Arabia riyal (SAR) (2399.6 (300.0–4199.2) United States dollar (USD)). The total median cost associated with hypoglycemia hospitalization was 4696.0 (886.5–12789.5) SAR (1252.0 (236.4–3410.0) USD). Ex-smokers were more likely to have higher hospitalization costs for hypoglycemia (4.4-folds) (*P* < .001). Being admitted to the ICU increased the likelihood of having a longer length of hospitalization by 2.6-folds (*P* < .001). Patients with longer diabetes duration (above 9 years) were more likely to be admitted to the ICU by 2.9-folds (*P* = .008). Understanding the factors that affect hypoglycemia hospitalization cost and length is essential for improving diabetes care and resource usage. Identifying high-risk patients and implementing efficient preventative strategies can lower the economic burden of DM and accompanying hypoglycemic episodes and enhance DM management.

## 1. Introduction

Diabetes mellitus (DM) is a common and potentially life-threatening medical condition, mostly attributed to its association with a range of severe chronic and acute outcomes.^[1]^ According to the most recent available statistics in 2021, the Kingdom of Saudi Arabia has a population of approximately 4.2 million individuals who have been diagnosed with diabetes mellitus with an estimated cost of $7.4 million.^[2]^ Expenditures related to diabetes mellitus encompass both direct and indirect medical costs, comprising hospital inpatient care, visits to the emergency department, diagnostic tests, and prescription drugs. Additionally, the economic impact includes the loss of productivity resulting from both morbidity and mortality.^[3]^

Hypoglycemia is a significant consequence of intensive antidiabetic therapy, often linked to the use of insulin, insulin secretagogues, and the overall intensity of the antidiabetic treatment.^[4–6]^ The identification and prevention of hypoglycemia might mitigate the consequences of diabetes by avoiding complications arising from hypoglycemia. Untreated severe hypoglycemia can result in significant financial and personal consequences.^[7–9]^ Individuals diagnosed with diabetes mellitus who encounter extreme hypoglycemia may undergo notable cognitive impairment, alongside life-threatening vascular incidents such as myocardial infarction, stroke, and cardiac arrhythmias, resulting in considerable economic consequences.^[10]^ Based on previous research, the prevalence of hypoglycemia in individuals with diabetes who are using oral medicines varies between 16% and 39%.^[11,12]^ Hypoglycemia was observed in around 45% of mild/moderate episodes and 6% of severe incidents.^[13]^ The financial implications of hypoglycemia episodes cannot be ignored,^[7,14–17]^ since they constitute a significant proportion of the overall expenses related to the management and treatment of diabetes mellitus.^[18]^ The estimation of costs related to hypoglycemia necessitates the computation of both direct and indirect medical expenses, which exhibit substantial variation contingent upon the severity of the hypoglycemic incident.^[19]^ The estimation of the financial consequences of hypoglycemia on healthcare services holds significant importance as it enables the formulation of initiatives aimed at improving diabetes management and enhancing the overall quality of patient care.^[20]^

Examining the economic impact of hypoglycemia among patients diagnosed with diabetes mellitus in Saudi Arabia is important due to the high prevalence of diabetes mellitus in the country.^[2]^ A previous study in Riyadh, Saudi Arabia examined the knowledge of hypoglycemia and its associated risk factors among type 2 diabetes mellitus patients in diabetes center in Security Forces Hospital. This study found that around 62.0% of the patients experienced hypoglycemic event in the last 3 months. Besides, around 47.0% of the patients experienced more than 3 hypoglycemic events. Another study in Alhasa, Saudi Arabia assessed the knowledge about hypoglycemia and its management among insulin-requiring diabetes mellitus patients.^[21]^ This study found that the majority of the patients (87.0%) had a good level of knowledge regarding hypoglycemia. At the same time, this study concluded that patients’ information about hypoglycemia complications and management was insufficient.^[21]^

Due to the high prevalence of diabetes mellitus in Saudi Arabia, investigating the economic burden of hypoglycemia has become increasingly important. Therefore, this study aimed to assess the length of stay and cost per hypoglycemia episode, as well as to determine the factors that influence the length of stay, intensive care unit (ICU) admission, and hospitalization costs among patients with diabetes mellitus in Saudi Arabia. It is imperative for healthcare practitioners to have a comprehensive understanding of the length of hospitalization and the corresponding expenses incurred by patients who experience hypoglycemia episodes. This information is crucial in order to optimize clinical treatment strategies. This contributes to the provision of adequate healthcare services to patients and the effective utilization of healthcare resources at the national level.

## 2. Methods

### 2.1. Study design

This is a retrospective cross-sectional study conducted on a cohort of individuals diagnosed with diabetes mellitus who experienced confirmed hypoglycemia episodes. The data pertaining to these patients were obtained from their respective hospital medical records, covering the period from January 2021 to December 2022.

### 2.2. Study population

The study population consisted of individuals who had received a diagnosis of either type 1 or type 2 diabetes and had sought medical care in the emergency department of King Fahad Medical City (KFMC), Riyadh, Saudi Arabia as a result of experiencing a hypoglycemic episode. The electronic medical records were used to identify patients who were hospitalized for hypoglycemia as their main cause of hospitalization.

### 2.3. Study site

KFMC is a 1200-bed facility that provides tertiary/quaternary healthcare services as part of the Riyadh Second Health Cluster under the Ministry of Health in the Kingdom of Saudi Arabia. The Medical City contains 4 hospitals and 4 specialized centers, and one of these centers is named Obesity, Endocrine and Metabolism Centre which provides care to individuals diagnosed with diabetes mellitus and obese patients in addition to patients with metabolic diseases in both inpatient and outpatient settings. One of the main services that KFMC also provides is Critical Care which is offered in 100 + beds of Intensive Care Units.

### 2.4. Data extraction

The data were acquired from the electronic database present at KFMC for each qualified patient. The data collected encompassed various aspects, including patient demographics, medical history, specific information related to DM such as the type and duration of the disease, as well as family history of DM. Additionally, the data included details about medication usage, including antidiabetic medications and other prescribed medications. Patient-specific clinical information, such as previous surgical history, engagement in daily exercises, living situation, and level of dependency in activities of daily living, was also documented. Furthermore, information pertaining to patient admission, such as behavior during admission, mental health status, communication barriers, admission time, length of stay, and discharge status, was documented. Health scores during admission, such as the Glasgow Coma Scale, venous thromboembolism risk factor score, and Braden risk assessment score, were also included in the data. Lastly, information specific to hypoglycemia events, such as the time of the event and the corresponding blood glucose level (measured in mg/dL) at the time of the event, was recorded. Furthermore, all pertinent data pertaining to the consumption of items throughout hospitalization resulting from hypoglycemia were extracted. This encompassed visits by physicians, expenses incurred for hospital stay, expenses incurred for ICU stay, prescriptions administered, laboratory tests requested, and radiological examinations ordered.

### 2.5. Study outcomes

In order to ascertain eligibility, an examination of the medical records of patients who were admitted to the hospital was conducted. According to the American Diabetes Association, hypoglycemia is defined as a condition where blood glucose level goes below 70 mg/dL (3.9 mmol/L).^[22]^ It is classified into 3 levels: mild (level 1), less than 70 mg/dL and greater than or equal to 54 mg/dL, usually requiring self-management; moderate (level 2), with levels below 54 mg/dL, clinically significant, and needs immediate treatment; and severe (level 3), very low levels causing significant cognitive impairment, needing external assistance for recovery, and is considered life-threatening with serious health risks when not treated.^[22]^. The research outcomes encompassed the financial implications of hospitalization, duration of hospital stay, and mortality rate. The mortality rate among patients admitted with hypoglycemia was determined by calculating the ratio of patients who died during their hospitalization to the total number of patients admitted. The estimation of the ICU admission rate was conducted by calculating the ratio of patients who were admitted to the ICU unit during their hospitalization to the overall number of admitted patients.

### 2.6. Costs estimation

Direct medical costs associated with controlling hypoglycemic episodes, including hospitalization and emergency room care, were identified.^[23]^ The financial department of KFMC reached out to request details regarding the charges associated with the care of specific patients. Hospitalization costs encompass various emergency management expenditures, including inpatient admission services, the expenses associated with healthcare personnel providing observation time, as well as the costs of medical treatments and medications provided, lab tests conducted, and radiology examinations.

The estimation of hospitalization expenses was conducted in Saudi Riyal (SAR) (each 1 SAR is equal to $0.27). All expenses were adjusted to reflect costs in the year 2023. The subsequent equation was employed to compute the inflated expenditure:


Inflated cost=Initial cost∗(1+inflation rate)^n 

Initial cost: the cost in any year before 2023; inflation rate: average inflation rate during the years of interest; n: number of years before 2023.

This study investigated the financial implications of hospitalization associated with hypoglycemia, with a specific focus on determining the average direct economic burden per patient for each hypoglycemic event. Indirect expenses, such as the impact on productivity resulting from absences from work, would be disregarded.

### 2.7. Ethical approval

Ethics approval was obtained for this study from the Research Ethics Committee at Isra University, Amman, Jordan (Project ID: PH-2020-14) and the Institutional Review Board at KFMC (H-01-*R*-012; IRB log Number 22-294).

### 2.8. Statistical analysis

The data analysis was conducted using the Statistical Package for Social Science application (version 27). In the case of variables that do not follow a normal distribution, the presentation of continuous data was carried out using the median and interquartile range (IQR). Categorical data was represented in the form of percentages and frequencies. Descriptive statistics were employed to characterize patient demographics, medication utilization, and comorbidities. The researchers employed binary logistic regression analysis to ascertain the factors that predict increased costs of hospitalization for hypoglycemia, longer durations of stay, and admission to the ICU. The level of statistical significance for the findings was indicated by a 95% confidence interval (*P* ≤ .05). The predetermined level of statistical significance was established at 5%.

## 3. Results

### 3.1. Patients’ baseline characteristics

Table 1 presents the baseline characteristics of the patients who were admitted for hypoglycemia. A total of 396 patients were involved in this study. Around 54.0% of them were males. The majority of them (76.3%) were single. More than half of them (55.8%) were current smokers. The mean body mass index for the patients was 20.2 (8.5) kg/cm^2^. The vast majority of them (92.9%) are diagnosed with type 2 diabetes mellitus. The mean duration of the disease was 9.0 (±3.4) years. A total of 8.8% of them reported that they have a family history of DM. Only 0.8% of the patients reported that they exercise regularly on a daily basis. Around one-fifth of the patients (20.2%) reported that they had previous surgery history.

**Table 1:** Patients’ baseline characteristics of the study participants

Variable*		
Gender		
Males	214	54.0
Mean age (±SD) years	33.6 (±94.9) years	
Marital status		
Single	302	76.3
Married	85	21.5
Widowed	4	1.0
Divorced	5	1.3
Smoking status		
None-smoker	114	28.8
Ex-smoker	61	15.4
Current smoker	221	55.8
Mean body mass index (BMI) (±SD) kg/cm^2^	20.2 (±8.5) kg/cm^2^	
Diabetes mellitus type		
Type 1 diabetes mellitus	29	7.1
Type 2 diabetes mellitus	367	92.9
Mean duration of disease (±SD) years	9 (±3.4) years	
Family history of diabetes mellitus (yes)	35	8.8
Engagement in exercise at least 30 minutes/daily (Yes)	3	0.8
Previous surgery (yes)	80	20.2

BMI = body mass index, SD = standard deviation.

* Data were presented as either frequency and percentages or mean and standard deviation (±SD).

### 3.2. Patients’ profile and health status upon admission

Table 2 presents patients’ health status upon admission. Around 7.3% of the patients had abnormal behavior during admission and 4.8% showed abnormal mental health status. Only 1.3% of the patients demonstrated communication barriers. The vast majority (85.1%) of the patients reported that they live with their family and are active in their daily living (99.2%). The mean Glasgow coma score for the patients was 12.6 (±3.9). The mean venous thromboembolism risk factor score was 1.2 (±0.5). The mean Braden risk assessment score was 11.4 (±3.4).

**Table 2:** Patients’ health status upon admission

Variable*		
Behaviour during admission		
Normal	367	92.7
Abnormal (agitation, confusion, hypoactive, or irritable)	29	7.3
Mental health		
Normal	377	95.2
Abnormal (psychiatric, dementia, or retarded)	19	4.8
Communication barrier		
None	391	98.7
Yes (blind, or intellectual disabilities)	5	1.3
Living status		
Live alone	59	14.9
Live with family	337	85.1
Activity of daily living		
Active	393	99.2
Bedridden	3	0.8
Mean Glasgow coma score (out of 15) (±SD)	12.6 (±3.9)	
Mean venous thromboembolism risk factor score (±SD	1.2 (±0.5)	
Mean Braden risk assessment score (±SD)	11.4 (±3.4)	

SD = standard deviation.

* Data were presented as either frequency and percentages or mean and standard deviation (±SD).

### 3.3. Patients’ disease history and medication use history

Table 3 presents patients’ disease history. The most common comorbidities among the patients were hypertension, heart disease, and asthma/ chronic obstructive pulmonary disease (COPD) with 30.3%, 25.8%, and 24.5%, respectively.

**Table 3:** Patients’ disease history

Variable	Frequency	Percentage
Hypertension	120	30.3
Heart disease	102	25.8
Asthma/chronic obstructive pulmonary disease	97	24.5
Nephropathy	84	21.5
Dyslipidaemia	51	13.4
Cancer	43	11.2
Neuropathy	44	11.1
Stroke	42	11.1
Myocardial infarction	41	10.4
Epilepsy	39	9.8
Eye problems	37	9.3
GERD/Acid reflux	37	9.3
Liver disease	35	8.8
Thyroid problems	32	8.1
Mental health issues	26	6.8
Depression/anxiety	22	5.6
Retinopathy	21	5.3
Arthritis	19	4.8
Foot problems	18	4.5
Tuberculosis	2	0.5

GERD = gastroesophageal reflux disease.

Table 4 presents patients’ antidiabetic agent use history. The most commonly used antidiabetic therapies among the patients were insulin-based therapy, insulin and metformin combination therapy, and metformin monotherapy with 25.8%, 5.1%, and 4.3%, respectively.

**Table 4:** Patients’ antidiabetic agents use history

Variable	Frequency	Percentage
Insulin based therapy	102	25.8
Insulin and metformin	20	5.1
Metformin	17	4.3
Sulfonylureas	14	3.5
Insulin and sulfonylurea	8	2.0
Insulin, sulfonylurea and metformin	3	0.8
DPP4I and metformin	2	0.5
Insulin, sulfonylurea and DPP4I	1	0.3
DPP4I and TZD	1	0.3
Metformin and sulfonylurea	1	0.3

DPP4I = Dipeptidyl peptidase-4 inhibitor, TZD = Thiazolidinedione.

Table 5 presents other medication use history. The most commonly used medications among the patients were diuretics, corticosteroids, and antiplatelet, with 22.5%, 20.2%, and 16.9%, respectively.

**Table 5:** Patients’ other medication use history

Variable	Frequency	Percentage
Diuretics	89	22.5
Corticosteroids	80	20.2
Antiplatelet	67	16.9
Calcium channel blocker	66	16.7
Statin	66	16.7
Ulcer treatment	65	16.4
B-blocker	61	15.4
Aspirin	56	14.1
Heparin	53	13.4
Antipsychotics	38	9.6
Anticonvulsant	36	9.1
ACE inhibitors	30	7.6
Neuropathy treatment	29	7.3
Anticoagulant (warfarin)	25	6.3
Antidepressant	24	6.1
Inhaled corticosteroids	22	5.6
Nitrates	20	5.1
ARB	15	3.8
Anxiolytic	11	2.8

ACE = angiotensin-converting enzyme, ARB = angiotensin receptor blockers.

### 3.4. Patients’ hypoglycemia profile

Table 6 presents patients’ hypoglycemia profile upon admission. The median glucose level on admission was 66.5 (47.0–94.5) mg/dL. The median duration of stay for the patients was 7.0 (2.0–16.0) days. Only 3.0% of the patients had a previous hypoglycemia admission history. Around 53.3% of the patients were admitted to the ICU. The median duration of ICU admission stay was 1.0 (0.0–1.0) days.

**Table 6:** Patients’ hypoglycemia profile

Variable*	Frequency	Percentage
Median glucose level on admission (mg/dL)	66.5 (47.0–94.5)	
Median duration of stay (d)	7.0 (2.0–16.0)	
Previously admitted for hypoglycemia (Yes)	12	3.0
Admitted to ICU (yes)	211	53.3
Median duration of ICU admission stay (days)	1.0 (0.0–1.0)	

ICU = intensive care unit, SD = standard deviation.

* Data were presented as either frequency and percentages or mean and standard deviation (±SD).

### 3.5. Costs of admissions due to hypoglycemia

Table 7 presents the costs of admissions due to hypoglycemia. The highest cost driver for patients with hypoglycemia was ICU stay with a median cost of 9000.0 (1125.0–15750.0) SAR (2399.6 (300.0–4199.2) United States dollar (USD)). The total median cost associated with hypoglycemia hospitalization was 4696.0 (886.5–12789.5) SAR (1252.0 (236.4–3410.0) USD).

**Table 7:** Costs of admissions due to hypoglycemia

Type of cost	Median cost (interquartile range)
Emergency department visit cost	300.0 (0–300.0) SAR (80.0(0–80.0) USD)
Physicians consultation cost	0 (0–750.0) SAR (0 (0–200.0) USD)
Inpatient admission stay cost	1267.0 (38.6–9315.0) SAR (337.8 (10.3–2483.6) USD)
Lab test cost	1057.0 (255.8–2353.0) SAR (281.8 (68.2–627.4) USD)
Radiological examination cost	0 (0–7266.0) SAR (0 (0–1937.3) USD)
ICU stay cost	9000.0 (1125.0–15,750.0) SAR (2399.6 (300.0–4199.2) USD)
Total cost	4696.0 (886.5–12,789.5) SAR (1252.0 (236.4–3410.0) USD)

ICU = Intensive care unit, SAR = Saudi Arabia Riyal, USD = United States dollar.

### 3.6. Predictors of hospitalization cost, duration of stay and ICU admission

Binary logistic regression analysis identified that ex-smokers were more likely to have higher hospitalization costs for hypoglycemia (4.4-folds) (*P* < .001). Being admitted to the ICU increased the likelihood of having a longer length of hospitalization by 2.6-folds (*P* < 0.001). Patients with longer diabetes duration (above 9 years) were more likely to be admitted to the ICU by 2.9-folds (*P* = .008, Table 8).

**Table 8:** Predictors of hospitalization cost, duration of stay, and ICU admission

Variable	Odds ratio of higher hospitalization cost (95% confidence interval)	*P* value		Odds ratio of longer length of hospitalization (95% confidence interval)	*P* value	Odds ratio for ICU admission (95% confidence interval)	*P* value	
Gender								
Females	1.00			1.00		1.00		
Males	1.19 (0.61–2.32)	.609		0.93 (0.63–1.38)	.719	1.39 (0.93–2.06)	.107	
Age category								
Below 33.6 years	1.00		1.00			1.00		
33.6 years and above	0.87 (0.42–1.81)	.708	1.15 (0.77–1.73)		.493	0.77 (0.52–1.16)		.215
Marital status								
Single	1.00			1.00		1.00		
Married	0.62 (0.24–1.64)	.337		1.20 (0.74–1.94)	.463	0.77 (0.48–1.25)	.293	
Widowed	–	–		0.93 (0.13–6.68)	.943	0.29 (0.03–2.80)	.284	
Divorced	–	–		0.62 (0.10–3.74)	.600	3.56 (0.39–32.10)	.258	
Smoking status								
None-smoker	1.00			1.00		1.00		
Ex-smoker	4.39 (2.04–9.41)	<.001		1.41 (0.81–2.46)	.219	0.71 (0.41–1.22)	.210	
Current smoker	0.171 (0.08–0.36)	<.001		0.98 (0.66–1.46)	.934	1.40 (0.94–2.09)	.095	
Body mass index category								
Below 20.2 kg/cm^2^	1.00		1.00			1.00		
20.2 kg/cm^2^ and above	0.69 (0.34–1.38)	.291	1.12 (0.75–1.67)		.593	1.05 (0.70–1.57)		.814
Diabetes mellitus type								
Type 1 diabetes mellitus	1.00			1.00		1.00		
Type 2 diabetes mellitus	4.13 (0.87–19.7)	.075		1.57 (0.75–3.30)	.236	1.42 (0.72–2.78)	.312	
Duration of disease category								
Below 9 years	1.00		1.00			1.00		
9 years and above	0.65 (0.17–2.40)	.514	1.32 (0.64–2.70)		.450	2.85 (1.32–6.17)		.008
Family history of diabetes mellitus								
No	1.00			1.00		1.00		
Yes	2.42 (0.80–7.39)	.119		0.52 (0.25–1.06)	.074	1.35 (0.67–2.74)	.406	
Engagement in exercise at least 30 minutes/daily								
No	1.00			1.00		1.00		
Yes	–	–		1.87 (0.17–20.81)	.610	1.76 (0.16–19.58)	.645	
Admitted to ICU								
No	1.00			1.00				
Yes	1.07 (0.53–2.13)	.859		2.56 (1.71–3.85)	<.001			
Previously admitted for hypoglycemia								
No	1.00			1.00		1.00		
Yes	1.42 (0.43–4.72)	.565		1.03 (0.99–1.07)	.171	0.98 (0.095–1.02)	.337	

ICU = intensive care unit.

## 4. Discussion

The results of this study suggest that hypoglycemia is a significant cost driver for patients with diabetes mellitus. This is in conformance with the study of Quilliam BJ et al who have conducted a retrospective study on 536,581 patients with hypoglycemic episodes that necessitate visiting the emergency department (ED), outpatient, or admitted as inpatients.^[24]^ Hypoglycemic episodes were associated with high per-episode costs with the mean costs for hypoglycemia visits being $17,564 for an inpatient admission, $1387 for an ED visit, and $394 for an outpatient visit. In a recent study published in Switzerland, researchers concluded that hypoglycemia led to a substantial socio-economic burden in Switzerland. Greater attention to non-severe hypoglycemic events and severe hypoglycemia in type 2 diabetes could have a major impact on reducing this burden.^[25]^ This considerable cost burden in terms of both medical resource consumption and productivity losses applies to severe and non-severe hypoglycemic episodes in the US,^[20]^ the Netherlands,^[26]^ the UK,^[27]^ Germany, and Spain.^[28]^ Failure to take into account this economic burden will devaluate the hypoglycemia risk mitigation policies.^[20]^ Although our study was restricted to exploring the cost of hospitalization due to hypoglycemia, the above studies indicate that hypoglycemia increases the cost burden during primary care visits, inpatient admissions as well as emergency room assessments.

In our study, the highest cost driver for patients with hypoglycemia was ICU stay, which is consistent with previous study. The longer duration of hospitalization and ICU admission were also associated with higher hospitalization costs. In a retrospective study on 3516 patients who required ED visits due to severe hypoglycemia in Italy,^[29]^ Veronese et al reported that 2426 patients (69%) were discharged from the ED while 31% were admitted.^[29]^ The total cost of the first group was around $583,785 compared to $6,588,173 for admitted patients. This gives a view as to the burden on the economy that hypoglycemia can incur. In addition, despite that 8.1% of the admitted patients were admitted to the ICUs, this small percentage incurred 35% of the total cost. This supports our findings that the ICU was the highest cost driver for patients with hypoglycemia was ICU stay with a median cost of 9000.0 SAR. The main driver of the cost of care of patients admitted to the ICU is the length of stay^[30]^ and avoidance of hypoglycemia significantly decreases the length of stay (LOS) and consequently cost of care in critically ill patients.^[31]^ In a multicentre international investigation, Krinsley et al^[31]^ demonstrated that, in critically ill patients, hypoglycemia was consistently associated with significantly higher ICU LOS regardless of severity. This increased LOS was significant and is likely to impact the magnitude of resource utilization.

Our study also found that ex-smokers were more likely to have higher hospitalization costs for hypoglycemia. This is likely due to the impact of smoking on the pancreas which may reduce insulin production.^[32]^ It was reported that smoking over time is associated with the development of the metabolic syndrome and diabetes mellitus.^[33]^ The association between smoking and severe hypoglycemia may be attributed to the effect of smoking on insulin clearance, which can result in hyperinsulinemia, an increased risk of postprandial hypoglycemia, and a deterioration of metabolic control.^[34]^ Also, Molla et al^[35]^ reported that smoking was associated with multiple diabetes mellitus complications including cardiovascular disease, neuropathy, nephropathy, and retinopathy and worse A1C and high-density lipoprotein control in both patients with type 1 and type 2 diabetes. On the other hand, studies have shown improvement in glycemic control after smoking cessation.^[36]^ Similar results were found comparing ex-smokers to nonsmokers. Former smokers can achieve similar glycemic control to nonsmokers and reduce the risk of diabetic complications.^[37]^

Patients with longer diabetes duration were also more likely to be admitted to the ICU, which may be attributed to other complications of diabetes, such as heart disease and stroke. Siegelaar et al,^[38]^ in their meta-analysis of 141 studies comprising 12,489,574 patients, including 2,705,624 deaths (21.7%) with at least 2,327,178 (18.6%) had diabetes, did not find any association between the presence of diabetes and mortality risk, however, they reported that critically ill patients with diabetes are at increased risk for the development of complications. It could be justified that the increased likelihood for patients in our study to be admitted to the ICU is due to long-term comorbidities associated with diabetes. Moreover, in a previous study conducted in Ethiopia, reported that longer duration of disease, comorbidities, chronic diabetes complications, higher blood glucose level, and treatment modality were significant determinants of hospital admission, readmission and longer hospital stay among T2D patients.^[39]^ Patients with diabetes mellitus and other co-existed conditions (such as myocardial ischemia, cardiac arrhythmia, stroke, dementia, and obesity) have higher odds of developing hypoglycemia compared to others.^[40]^ Patients diagnosed with diabetes mellitus have altered immunologic and inflammatory responses and metabolism make them more vulnerable to complications and hospitalization.^[40]^

Hypoglycemia unawareness is one of the main contributing factors that lead to hypoglycemia. Around 10% to 25% of individuals diagnosed with diabetes mellitus experience hypoglycemia unawareness; in which they demonstrate diminished or no ability to perceived the onset of hypoglycemia.^[41]^ One of the main risk factors for hypoglycemia unawareness is older age and longer disease duration (having diabetes mellitus for 20–30 years). Besides, patients with mental disorders demonstrate diminished ability to recognize and manage hypoglycemic episodes.^[41]^ It has been demonstrated that patient education can enhance the management of hypoglycemia.^[42]^ It is imperative to conduct routine reviews of medications that are susceptible to hypoglycemia with patients and their families.^[42]^ The recognition and management of hypoglycemia symptoms should be addressed during each visit. Additionally, written instructions should be provided to all patients, as caregivers may become overwhelmed and forget the steps to reverse hypoglycemia in an emergency. It is recommended that patients inform their clinicians of any hypoglycemic episodes they are experiencing in order to modify their treatment regimens (mode of therapy) as necessary.^[42]^

In our study, upon admission, some patients exhibited abnormal behavior (7.3%) and abnormal mental health status (4.8%), while only 1.3% had communication barriers. The majority of patients lived with their families (85.1%) and were active in their daily lives (99.2%). Including mental health as part of a multidisciplinary team to manage diabetes mellitus may be worthwhile according to these results. Amrit Sachar reported that addressing psychological and mental issues like depression, anxiety, eating disorders, cognitive impairment, interpersonal difficulties, and personality disorders can improve health and financial outcomes.^[43]^ High rates of mental health problems in diabetes are an obstacle to effective self-management and proposed that mental health be integrated into diabetes care protocols to provide effective treatment for diabetes and to improve confidence among healthcare professionals who support individuals diagnosed with diabetes mellitus.^[43]^ In line with this recommendation, Young et al emphasized similar recommendations in their position statement on the psychosocial status of people with diabetes.^[23]^ They recommended that individuals diagnosed with diabetes mellitus be screened periodically for depression, stress, dietary issues, and anxiety. In addition, they suggested to include behavioral health services in the diabetes management team.

In our study, regarding patients’ disease history and medication use, the most common comorbidities were hypertension, heart diseases, and asthma/COPD. These comorbidities could potentially complicate diabetes mellitus treatment and add to the risk of hypoglycemia during hospitalization.^[44]^ Using UK Clinical Practice Research Datalink (CPRD) linked with the Index of Multiple Deprivation data for the period between 2007 and 2017, Magdalena Nowakowska et al, on a sample of 102, 394 type 2 diabetes mellitus patients found that 32.6% of the patients have one or more comorbid conditions while 2.9% (in affluent areas) to 4.4% (in deprived areas) had 4 or more comorbidities.^[45]^ In support of our study, hypertension was the most prevalent comorbidity with a prevalence rate of 42.8% in males compared to 45.8% in females. As in our results, coronary heart disease was the second most prevalent comorbidity in male patients with a percentage of 13.6%, however, the second most prevalent disease among female type 2 diabetes mellitus patients was depression.^[45]^ In our study the prevalence of depression/anxiety was 5.6%, however, we did not stratify comorbidity findings between males and females which is worth looking at.

In our study, the most commonly used antidiabetic therapies were insulin-based therapy which is consistent with the fact that insulin remains a cornerstone in DM management. Additionally, diuretics, corticosteroids, and antiplatelet medications were commonly used among the patients. These medications are frequently used in the management of common DM comorbidities. It is clear that the order of therapy for type 2 diabetes mellitus differs with different experiences, schools and concomitant diseases. In a recent study in Colombia, Manuel E Machado-Duque et al, reported in 14,722 patients with type 2 diabetes mellitus and chronic kidney disease, insulin was used by almost 50% of the patients (pooled results of all kinds of insulin) followed by metformin, DPP-4 inhibitors, SGLT-2 inhibitors, GLP-1 analogues and finally sulfonylureas.^[46]^ The combination of therapy is also different e.g., 13.4% of our patients used a combination of DPP4 inhibitors and metformin while in this study only 0.5% of the patients used the same combination.^[46]^

Our study results demonstrated that the highest cost driver for hypoglycemia hospitalization was ICU stay, with a median cost of SAR 9000 [IQR 1125–15,750] (2399.6 (300.0–4199.2) USD). The total median cost of hypoglycemia hospitalization was SAR 4696 [IQR 886.5–12,789.5] (1252.0 (236.4–3410.0) USD). Furthermore, we conducted a binary logistic regression analysis to identify predictors of hospitalization cost, duration of stay, and ICU admission. Ex-smokers were found to have 4.4 times higher hospitalization costs (*P* < .001). Being admitted to the ICU increased the likelihood of a longer hospitalization stay by 2.6 times (*P* < .001) which indicates the importance of preventing severe hypoglycemia events. Patients with longer diabetes duration (above 9 years) need closer monitoring by a multidisciplinary team approach as they were more likely to be admitted to the ICU by 2.9 times (*P* = .008), possibly due to comorbidities. Being a current smoker was associated with a significantly lower likelihood of higher hospitalization costs (*P* < .001) which was likely because current smokers were significantly younger than ex-smokers.

In our study, length of stay did not have a statistically significant connection to certain demographic and clinical factors such as age, marital status, and body mass index. Conversely, the odds of longer hospitalization significantly increased by ICU admission, which indicates a possible impact of severe hypoglycemia on hospital length of stay. Similarly, patients who had diabetes for longer durations were more likely to require ICU admissions, suggesting that this subpopulation might be at higher risk of hypoglycemia-related complications.

Understanding the factors affecting the cost and length of hospitalization of hypoglycemia in individuals diagnosed with diabetes mellitus is crucial for developing strategies to improve diabetes care and optimize resource utilization at the national level. Due to the high prevalence of diabetes mellitus in Saudi Arabia,^[2]^ it becomes necessarily to give more attention toward understanding the economic impact of hypoglycemia as one of the main adverse events associated with antidiabetic medications, in order to implement actions that diminish its financial consequences. Furthermore, studying LOS associated with hypoglycemic events and its associated predictors provides healthcare professionals and decision-makers with an opportunity to decrease its associated indirect costs that might emerge from the loss of productivity. Healthcare systems can potentially reduce the economic burden of diabetes mellitus and its related hypoglycemic episodes by identifying high-risk patients and implementing effective preventive measures that can also improve the overall quality of care for individuals with diabetes mellitus.

The limitations of the current study include its retrospective cross-sectional design, which makes establishing causal relationships between variables challenging. The study was also conducted in a single center which limits the generalisability of the results to other settings or regions. Besides, there is no previous research in Saudi Arabia that examined cost of hypoglycemia among patients hospitalized for hypoglycemia; which restricted our ability to compare our study findings to other studies in Saudi Arabia. Employing a prospective study design and including a more diverse patient population is advised to overcome these limitations in future research.

In conclusion, our study sheds light on the economic burden of hypoglycemia management among individuals diagnosed with diabetes mellitus. The findings of our study suggest that hospitalization costs and length of hospital stay may be effectively managed by preventing severe hypoglycemic events, improving disease management, and addressing comorbidities. Additionally, factors such as smoking history and disease duration were shown to have a possible influence on the burden of hypoglycemia-related hospitalizations.

## Acknowledgments

The authors would like to thank the Research Centre at King Fahd Medical City, Riyadh Second Health Cluster, Riyadh, for their valuable technical support provided for the manuscript.

## Author contributions

**Conceptualization:** Abdallah Y. Naser, Hind M. AlOsaimi, Haya bin huwayshil.

**Data curation:** Abdallah Y. Naser.

**Formal analysis:** Abdallah Y. Naser.

**Funding acquisition:** Hind M. AlOsaimi.

**Investigation:** Abdallah Y. Naser, Sami Qadus, Hind M. AlOsaimi, Abdulrahman AlFayez, Haya bin huwayshil, Lujain A. Al Harbi, Malak S. Alqhtani, Nayef A. Alyamani.

**Methodology:** Abdallah Y. Naser, Hind M. AlOsaimi.

**Project administration:** Abdallah Y. Naser, Hind M. AlOsaimi.

**Resources:** Sami Qadus, Hind M. AlOsaimi, Haya bin huwayshil, Lujain A. Al Harbi, Malak S. Alqhtani, Nayef A. Alyamani.

**Software:** Abdallah Y. Naser.

**Supervision:** Abdallah Y. Naser, Sami Qadus, Hind M. AlOsaimi.

**Validation:** Abdallah Y. Naser, Hind M. AlOsaimi.

**Visualization:** Abdallah Y. Naser, Hind M. AlOsaimi.

**Writing – original draft:** Abdallah Y. Naser, Sami Qadus, Hind M. AlOsaimi, Abdulrahman AlFayez, Haya bin huwayshil, Lujain A. Al Harbi, Malak S. Alqhtani, Nayef A. Alyamani.

**Writing – review & editing:** Abdallah Y. Naser, Sami Qadus, Hind M. AlOsaimi, Abdulrahman AlFayez, Haya bin huwayshil, Lujain A. Al Harbi, Malak S. Alqhtani, Nayef A. Alyamani.

## References

[1] Collaborators, G.B.D.D. Global, regional, and national burden of diabetes from 1990 to 2021, with projections of prevalence to 2050: a systematic analysis for the Global Burden of Disease Study 2021. Lancet. 2023;402:203–34. 37356446 10.1016/S0140-6736(23)01301-6 PMC10364581

[2] International Diabetes Federation. Saudi Arabia: Diabetes report 2000 – 2045. 2023 September 16, 2023]. https://diabetesatlas.org/data/en/country/174/sa.html. Accessed January 15, 2024.

[3] Ng CS Lee JY Toh MP Ko Y. Cost-of-illness studies of diabetes mellitus: a systematic review. Diabetes Res Clin Pract. 2014;105:151–63. 24814877 10.1016/j.diabres.2014.03.020

[4] Umpierrez G Korytkowski M. Diabetic emergencies - ketoacidosis, hyperglycaemic hyperosmolar state and hypoglycaemia. Nat Rev Endocrinol. 2016;12:222–32. 26893262 10.1038/nrendo.2016.15

[5] Naser AY Wong ICK Whittlesea C. Use of multiple antidiabetic medications in patients with diabetes and its association with hypoglycaemic events: a case-crossover study in Jordan. BMJ Open. 2018;8:e024909. 10.1136/bmjopen-2018-024909 PMC6252777 30467136

[6] Naser AY Alsairafi Z Alwafi H. The perspectives of physicians regarding antidiabetic therapy de-intensification and factors affecting their treatment choices-A cross-sectional study. Int J Clin Pract. 2021;75:e13662. 32770843 10.1111/ijcp.13662

[7] Miller GHC Byington ME Goff RP.; Action to Control Cardiovascular Risk in Diabetes Study Group, Effects of intensive glucose lowering in type 2 diabetes. New England J Med. 2008;358: 2545–59. 18539917 10.1056/NEJMoa0802743 PMC4551392

[8] Alwafi H Alsharif AA Wei L. Incidence and prevalence of hypoglycaemia in type 1 and type 2 diabetes individuals: a systematic review and meta-analysis. Diabetes Res Clin Pract. 2020;170:108522. 33096187 10.1016/j.diabres.2020.108522

[9] Naser AY Wang Q Wong LYL. Hospital admissions due to dysglycaemia and prescriptions of antidiabetic medications in England and Wales: an ecological study. Diabetes Ther. 2018;9:153–63. 29260459 10.1007/s13300-017-0349-1 PMC5801235

[10] Frier BM Schernthaner G Heller SR. Hypoglycemia and cardiovascular risks. Diabetes Care. 2011;34:S132–7. 21525444 10.2337/dc11-s220 PMC3632150

[11] Chan SP Ji LN Nitiyanant W Baik SH Sheu WH. Hypoglycemic symptoms in patients with type 2 diabetes in Asia-Pacific-Real-life effectiveness and care patterns of diabetes management: the RECAP-DM study. Diabetes Res Clin Pract. 2010;89:e30–2. 20541826 10.1016/j.diabres.2010.05.008

[12] Miller CD Phillips LS Ziemer DC. Hypoglycemia in patients with type 2 diabetes mellitus. Arch Intern Med. 2001;161:1653–9. 11434798 10.1001/archinte.161.13.1653

[13] Edridge CL Dunkley AJ Bodicoat DH. Prevalence and incidence of hypoglycaemia in 532,542 people with type 2 diabetes on oral therapies and insulin: a systematic review and meta-analysis of population based studies. PLoS One. 2015;10:e0126427–20. 26061690 10.1371/journal.pone.0126427 PMC4465495

[14] Liu S Zhao Y Hempe JM Fonseca V Shi L. Economic burden of hypoglycemia in patients with type 2 diabetes. Expert Rev Pharmacoecon Outcomes Res. 2012;12:47–51. 22280196 10.1586/erp.11.87

[15] McEwan P Larsen Thorsted B Wolden M Jacobsen J Evans M. Healthcare resource implications of hypoglycemia-related hospital admissions and inpatient hypoglycemia: retrospective record-linked cohort studies in England. BMJ Open Diabetes Res Care. 2015;3:e000057–6. 10.1136/bmjdrc-2014-000057 PMC4368913 25815204

[16] Heller SR Frier BM Hersløv ML Gundgaard J Gough SC. Severe hypoglycaemia in adults with insulin-treated diabetes: impact on healthcare resources. Diabetic Med. 2016;33:471–7. 26179360 10.1111/dme.12844 PMC5034744

[17] Naser AY Alwafi H Alsairafi Z. Cost of hospitalisation and length of stay due to hypoglycaemia in patients with diabetes mellitus: a cross-sectional study. Pharmacy Pract. 2020;18:1847–8. 10.18549/PharmPract.2020.2.1847 PMC7290179 32566047

[18] Harris SB Leiter LA Yale J-F Chiasson J-L Kleinstiver P Sauriol L. Out-of-pocket costs of managing hyperglycemia and hypoglycemia in patients with type 1 diabetes and insulin-treated type 2 Diabetes. Can J Diabetes. 2007;31:25–33.

[19] Kim G Lee YH Han MH. Economic burden of hypoglycemia in patients with type 2 Diabetes Mellitus from Korea. PLoS One. 2016;11:1–11. 10.1371/journal.pone.0151282 PMC4790854 26975054

[20] Foos V Varol N Curtis BH. Economic impact of severe and non-severe hypoglycemia in patients with Type 1 and Type 2 diabetes in the United States. J Med Econ. 2015;18:420–32. 25629654 10.3111/13696998.2015.1006730

[21] Al Hussaini H Alismael A Alquraini M. Knowledge Regarding Hypoglycemia and Its Management Among Patients With Insulin-Requiring Diabetes Mellitus in Al-Ahsa, Saudi Arabia. Cureus. 2023;15:e47257. 37859676 10.7759/cureus.47257 PMC10583613

[22] American Diabetes Association Professional Practice Committee. 6. Glycemic Goals and Hypoglycemia: Standards of Care in Diabetes-2024. Diabetes Care. 2024;47(Suppl 1):S111–s125. 38078586 10.2337/dc24-S006 PMC10725808

[23] Young-Hyman D de Groot M Hill-Briggs F Gonzalez JS Hood K Peyrot M. Psychosocial care for people with diabetes: a position statement of the American Diabetes Association. Diabetes Care. 2016;39:2126–40. 27879358 10.2337/dc16-2053 PMC5127231

[24] Quilliam BJ Simeone JC Ozbay AB Kogut SJ. The incidence and costs of hypoglycemia in type 2 diabetes. Am J Manag Care. 2011;17:673–80. 22106460

[25] Tzogiou C Wieser S Eichler K. Incidence and costs of hypoglycemia in insulin-treated diabetes in Switzerland: a health-economic analysis. J Diabetes Complications. 2023;37:108476. 37141836 10.1016/j.jdiacomp.2023.108476

[26] de Groot S Enters-Weijnen CF Geelhoed-Duijvestijn PH Kanters TA. A cost of illness study of hypoglycaemic events in insulin-treated diabetes in the Netherlands. BMJ Open. 2018;8:e019864–10. 10.1136/bmjopen-2017-019864 PMC5875590 29581204

[27] Parekh WA Ashley D Chubb B Gillies H Evans M. Approach to assessing the economic impact of insulin-related hypoglycaemia using the novel Local Impact of Hypoglycaemia Tool. Diabetic Med. 2015;32:1156–66. 25816891 10.1111/dme.12771 PMC5029754

[28] Hammer M Lammert M Mejías SM Kern W Frier BM. Costs of managing severe hypoglycaemia in three European countries. J Med Econ. 2009;12:281–90. 20001570 10.3111/13696990903336597

[29] Veronese G Marchesini G Forlani G.; Italian Society of Emergency Medicine (SIMEU). Costs associated with emergency care and hospitalization for severe hypoglycemia. Nutr Metab Cardiovasc Dis. 2016;26:345–51. 26897390 10.1016/j.numecd.2016.01.007

[30] Dasta JF McLaughlin TP Mody SH Piech CT. Daily cost of an intensive care unit day: the contribution of mechanical ventilation. Crit Care Med. 2005;33:1266–71. 15942342 10.1097/01.ccm.0000164543.14619.00

[31] Krinsley J Schultz MJ Spronk PE. Mild hypoglycemia is strongly associated with increased intensive care unit length of stay. Ann Intensive Care. 2011;1:1–9. 22115519 10.1186/2110-5820-1-49 PMC3273438

[32] Śliwińska-Mossoń M Milnerowicz H. The impact of smoking on the development of diabetes and its complications. Diab Vasc Dis Res. 2017;14:265–76. 28393534 10.1177/1479164117701876

[33] Kondo T Nakano Y Adachi S Murohara T. Effects of tobacco smoking on cardiovascular disease. Circ J. 2019;83:1980–5. 31462607 10.1253/circj.CJ-19-0323

[34] Hirai FE Moss SE Klein BEK Klein R. Severe hypoglycemia and smoking in a long-term type 1 diabetic population: wisconsin epidemiologic study of diabetic retinopathy. Diabetes Care. 2007;30:1437–41. 17372156 10.2337/dc06-2264

[35] Molla GJ Ismail-Beigi F Larijani B. Smoking and diabetes control in adults with type 1 and type 2 diabetes: a nationwide study from the 2018 national program for prevention and control of diabetes of Iran. Can J Diabetes. 2020;44:246–52. 31494031 10.1016/j.jcjd.2019.07.002

[36] Walicka M Russo C Baxter M John I Caci G Polosa R. Impact of stopping smoking on metabolic parameters in diabetes mellitus: a scoping review. World J Diabetes. 2022;13:422–33. 35800409 10.4239/wjd.v13.i6.422 PMC9210544

[37] Braffett BH Rice MM Young HA Lachin JM. Mediation of the association of smoking and microvascular complications by glycemic control in type 1 diabetes. PLoS One. 2019;14:e0210367–14. 30615671 10.1371/journal.pone.0210367 PMC6322792

[38] Siegelaar SE Hickmann M Hoekstra JBL Holleman F DeVries JH. The effect of diabetes on mortality in critically ill patients: a systematic review and meta-analysis. Crit Care. 2011;15:1–12. 10.1186/cc10440 PMC3334749 21914173

[39] Regassa LD Tola A. Magnitude and predictors of hospital admission, readmission, and length of stay among patients with type 2 diabetes at public hospitals of Eastern Ethiopia: a retrospective cohort study. BMC Endocrine Disorders. 2021;21:1–13. 33866969 10.1186/s12902-021-00744-3 PMC8054433

[40] Kong AP Chan JC. Hypoglycemia and comorbidities in type 2 diabetes. Curr Diab Rep. 2015;15:80. 26338288 10.1007/s11892-015-0646-x

[41] National Institute of Diabetes and Digestive and Kidney Diseases. How Hypoglycemia Unawareness Affects People with Diabetes. 2024. https://www.niddk.nih.gov/health-information/professionals/diabetes-discoveries-practice/how-hypoglycemia-unawareness-affects-people-with-diabetes. Accessed April 5, 2023.

[42] Unger J. Educating patients about hypoglycemia prevention and self-management. Clin Diabetes. 2013;31:179–88.

[43] Sachar A. How important is mental health involvement in integrated diabetes care? The Inner North West London experience. London J Primary Care. 2012;5:63–7. 10.1080/17571472.2013.11493377 PMC4413702 25949671

[44] Silbert R Salcido-Montenegro A Rodriguez-Gutierrez R Katabi A McCoy RG. Hypoglycemia among patients with type 2 Diabetes: epidemiology, risk factors, and prevention strategies. Curr Diab Rep. 2018;18:1–27. 29931579 10.1007/s11892-018-1018-0 PMC6117835

[45] Nowakowska M Zghebi SS Ashcroft DM. The comorbidity burden of type 2 diabetes mellitus: patterns, clusters and predictions from a large English primary care cohort. BMC Med. 2019;17:1–10. 31345214 10.1186/s12916-019-1373-y PMC6659216

[46] Machado-Duque ME Gaviria-Mendoza A Valladales-Restrepo LF. Treatment patterns of antidiabetic and kidney protective therapies among patients with type 2 diabetes mellitus and chronic kidney disease in Colombia. The KDICO descriptive study. Diabetol Metab Syndrome. 2023;15:1–11. 10.1186/s13098-023-01126-6 PMC10318702 37403118

